# Comparison of the effects of occlusal splint and Botox injections on the amount of mouth opening and chronic pain in individuals with temporomandibular disorders: a systematic review and meta‐analysis

**DOI:** 10.1111/adj.13059

**Published:** 2025-02-14

**Authors:** Ö İşisağ, H Atasoy, S Yıldız

**Affiliations:** ^1^ Department of Prosthodontics, Faculty of Dentistry Afyonkarahisar Health Sciences University Afyonkarahisar Turkey

**Keywords:** Temporomandibular joint disorders, occlusal splints, botulinum toxins

## Abstract

**Objective:**

This systematic review and meta‐analysis study was carried out to compare the effectiveness of occlusal splint (OS) and Botox (BTX) injections in temporomandibular disorders (TMDs).

**Material and Methods:**

Irrespective of the starting year, studies were searched for up to 2024 using PubMed, Web of Science, Scopus databases and the Google Scholar search engine. In the study, graded chronic pain scale and maximum mouth opening amount parameters were analysed with Comprehensive Meta‐Analysis programme.

**Results:**

Out of 619 studies, only six were included in the meta‐analysis. The effect size was 0.293 in favour of BTX in the studies evaluating the maximum mouth‐opening (MMO) range. However, the amount of MMO did not show a statistically significant difference between the OS and BTX groups (95% CI – 0.383 to 0.969, *P* = 0.395, *z* = −0.850). In the subgroup analyses carried out based on the follow‐up periods, it was found that the MMO was statistically significantly higher in the BTX group at the first week and in the OS group at the third month. However, there was no significant difference observed at the first, second, sixth and twelfth month follow‐ups (*P* > 0.05). No significant difference was found between the groups (*P* > 0.05) in the publications that evaluated the graded chronic pain scale (GCPS), although an effect size of 0.673 was found in favour of OS (95% CI 0.331 to 1.365, *P* = 0.272, *z* = −1.098).

**Conclusion:**

BTX injections and OS applications show significant differences in the MMO of individuals in the early period. In contrast, the MMO of individuals and the GCPS show similar findings in the sixth month and longer follow‐up.

**Abbreviations and acronyms**
BTX: Botox
CMA: Comprehensive Meta‐Analysis
GCPS: graded chronic pain scale
MMO: maximum mouth‐opening
OS: occlusal splint
TMDs: temporomandibular disorders

**Clinical Relevance**

According to the results of this study, BTX injections were found to be more effective than OS in the early stages of TMD, and both splints and BTX injections had similar effects on the amount of mouth opening and chronic pain in the long term. These findings might be useful for clinicians to consider when treating these disorders.

## INTRODUCTION

Temporomandibular disorders (TMDs) are musculoskeletal conditions affecting the temporomandibular joint (TMJ), masticatory system and related tissues. Although the aetiology of TMD is unclear, it might emerge due to several factors such as occlusal conditions, trauma, emotional stress, deep source of pain and parafunctional activity. TMD might be associated with the joint or masticatory muscles and can be treated with medical treatment, physiotherapy, surgical interventions, trigger point injections, acupuncture, OS or BTX injections.^1^, ^2^, ^3^, ^4^, ^5^, ^6^

OS are devices that relieve the pain observed in TMD and can bring the restricted mouth opening to its normal position. These devices interrupt the neuromuscular reflex contraction cycle, relax muscles in patients with parafunctional habits and help to increase occlusal stability while reducing muscle tension. They also protect the teeth against tooth surface wear and reposition the jaws in a centric relation. In the literature, various types of OS have been reported for the treatment of TMD. In addition to the most commonly used stabilization splints such as the Tanner apparatus, Fox apparatus, Michigan splint, or centric relation apparatus, anterior reposition splints and anterior bite plates are used in the treatment of TMD.^7^, ^8^, ^9^

Botulinum toxin A, known as BTX, is a neurotoxin obtained from the bacterium Clostridium botulinum. By interrupting the neurotransmission of acetylcholine delivered to the muscles through botox injection, treatment of various medical conditions associated with muscle spasms and hyperactivity can be ensured. BTX injections are also commonly used for cosmetic purposes such as reshaping the mandibular angle and treating masseter hypertrophy. Studies have shown the efficiency of botulinum toxin injections in the treatment of conditions such as TMD and bruxism as well as myofascial pain and muscle hyperactivity. From this point of view, BTX injections are used as an alternative to traditional treatments in the treatment of TMD.^10^, ^11^, ^12^, ^13^, ^14^

In the literature, studies examining the effects of OS and botulinum toxin injections on TMD reveal that both types of treatment alleviate the pain associated with TMD and increase the amount of mouth opening.^15^, ^16^, ^17^ However, no systematic review or meta‐analysis study comparing the effectiveness of these treatments postoperatively has been found in the literature. This meta‐analysis study was carried out to compare the effects of OS applications and BTX injections, which are frequently used in the treatment of individuals with TMD, on individuals' chronic pain conditions and MMO amounts. The null hypothesis of the study argued that both treatment processes would not show a statistically significant difference in terms of both mouth opening and chronic pain.

## MATERIALS AND METHODS

This systematic review and meta‐analysis study was carried out and reported in line with the Preferred Reporting Items for Systematic Reviews and Meta‐Analyses (PRISMA, 2020).

### Literature review

The online literature review was carried out by using PubMed, Web of Science, Scopus databases and Google Scholar search engine in a way to cover the studies until 2024 regardless of the starting year. The review strategy was implemented on PubMed, Web of Science and Scopus databases by using Boolean operators as follows: (“temporomandibular disorder” OR “patient”) AND (“stabilization splint” OR “occlusal splint”) AND (“botox” OR “botulinum toxin”) AND (“masseter pain” OR “bruxism”). As Boolean operators were not used in the Google Scholar search engine, searching was performed by entering the terms temporomandibular disorder, stabilization splint, botox and masseter pain in the advanced search section of the tab ‘with all of the words’ and patient, occlusal splint, botulinum toxin and bruxism in the tab ‘with at least one of the words’. The literature review was carried out on all databases and in the Google Scholar search engine with terms anywhere in the article. The articles obtained from Pub Med, Web of Science and Scopus databases were transferred to the Rayyan program used for systematic reviews and meta‐analysis studies, and the articles obtained from Google Scholar were manually examined.

Inclusion and exclusion criteria of the study:

Inclusion criteria:

1. Clinical follow‐up studies comparing the effectiveness of BTX and OS.
2. Studies written in English.

Exclusion criteria:

1. Clinical follow‐ups where botulinum toxin and occlusal splints were not included in the same study.
2. Non‐English studies.
3. Case reports, books, book sections, dissertations, reviews, systematic reviews and meta‐analyses, clinical protocols and clinical guidelines.
4. Studies whose full texts could not be accessed.

Data such as the first author, publication year, participant number, ages and genders, study groups and the interventions applied, follow‐up periods and outputs obtained were extracted from the studies included in the meta‐analysis. The risks of bias in the included studies were evaluated by three researchers in the light of the parameters given in Table 1.

**Table 1:** Data obtained from studies evaluating maximum mouth opening (MMO)

Author, year	Mean age ± SD (Gender)	Intervention (Sample size)	Follow‐up period	MMO
Shahine 2013^24^	30.58 ± 6.23 (Women)	BTX (n = 12) 30 U to masseter muscle, 25 U to temporal muscle Botox, Allergan injected (Three injection sites in each muscle during a single appointment) OS (n = 12) The splint prepared from hard acrylic resin in the maxillary arch was used for 12 weeks during sleep.	2 months	BTX: 30.0 ± 3.3 OS: 29.6 ± 1.8
			3 months	BTX: 37.3 ± 5.6 OS: 32.6 ± 3.1
Hong 2020^25^	26.9 ± 6.0 (Women)	BTX (n = 11) 20 U temporal muscle, 25 U masseter muscle injected with Botox, Allergan (Two sets of injections, with the first injection being administered the first time and the second one being administered at 6 months) OS (n = 12) The splint prepared from 2 mm thick acrylic resin in the maxillary arch was used for at least 8 hours a day for the duration of the treatment.	12 months	BTX:47.7 ± 4.9 OS:47.4 ± 5.9
Hong 2020^25^	55.3 ± 6.3 (Women)	BTX (n = 12) 20 U temporal muscle, 25 U masseter muscle injected with Botox, Allergan (Two sets of injections, with the first injection being administered the first time and the second one being administered at 6 months) OS (n = 10) The splint prepared from 2 mm thick acrylic resin in the maxillary arch was used for at least 8 hours a day for the duration of the treatment.	12 months	BTX:48.3 ± 4 OS:50.6 ± 3.5
Fathy 2020^26^	Between 20 and 40 years (Gender not available)	BTX (n = 20) BTX was injected into temporal and masseter muscles (dose information and number of injections not available) OS (n = 20) Used in the maxillary arch (duration of use not available)	1 week	BTX:21 ± 3.9 OS:37 ± 3.9
			3 months	BTX:44.3 ± 3.85 OS:39.2 ± 4.27
			6 months	BTX:37.4 ± 3.69 OS:39.2 ± 3.94
Hosgor 2023^27^	34.63 ± 11.85 (49 Women, 11 Men)	BTX (n = 30) 200 IU temporal muscle and 300 IU masseter muscle injected with Dysport (The frequency of injections or the number of sessions is unclear) OS (n = 30) A splint made of hard acrylic in the maxillary arch was used during sleep.	1 months	BTX:42.53 ± 6.06 OS:41.76 ± 5.86
			3 months	BTX:43.16 ± 5.56 OS:42.06 ± 5.75
			6 months	BTX:42.23 ± 6.48 OS:42.5 ± 4.92

### Statistical evaluation

All statistical analyses were performed in the Comprehensive Meta‐Analysis (CMA) software program at a significance level of *P* = 0.05. In addition, the standardized mean differences (SMD) and 95% confidence intervals (CI) of all included studies were calculated. A sensitivity analysis was carried out to evaluate the stability of the results by excluding one study every time. To evaluate statistical heterogeneity between the studies, heterogeneity was accepted at *P* < 0.1 values by using Cochran's Q statistics. Finally, subgroup analyses were carried out based on clinical follow‐up periods to evaluate the potential causes of heterogeneity.

## RESULTS

**Fig. 1:**
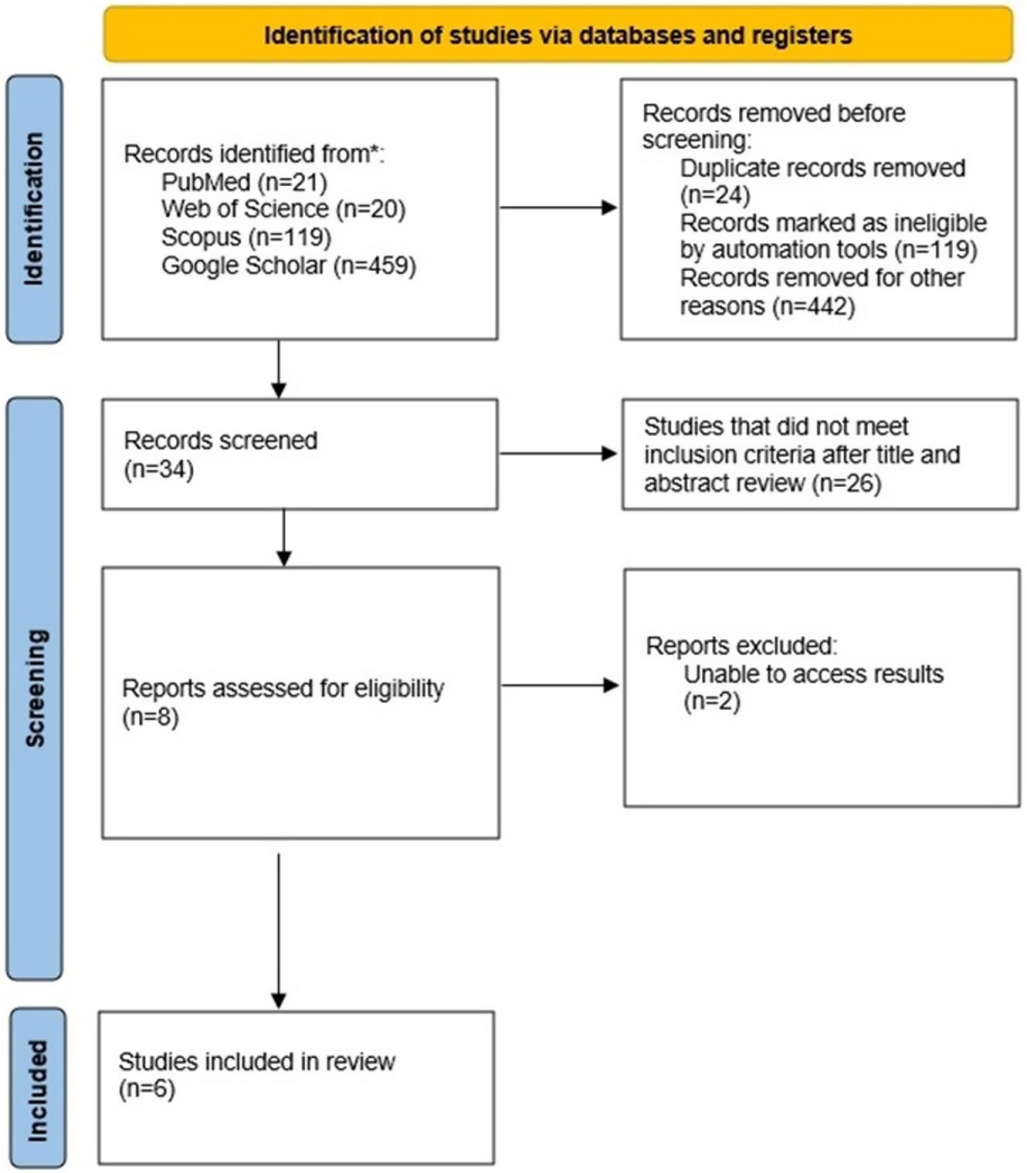
Identification of studies through databases and registries.

From PubMed, Web of Science and Scopus databases, 160 publications were accessed. Twenty‐four of them were excluded from the study due to the presence of at least on two of the scanned databases. The titles and abstracts of the remaining 136 articles were examined, 17 articles were examined further and 4 studies were included in the meta‐analysis. A total of 459 publications were accessed on the Google Scholar search engine, 17 articles were further examined and 2 publications were included in the study. As a result, 6 publications were included in the meta‐analysis from all databases and Google Scholar (Fig. 1).

**Fig. 2:**
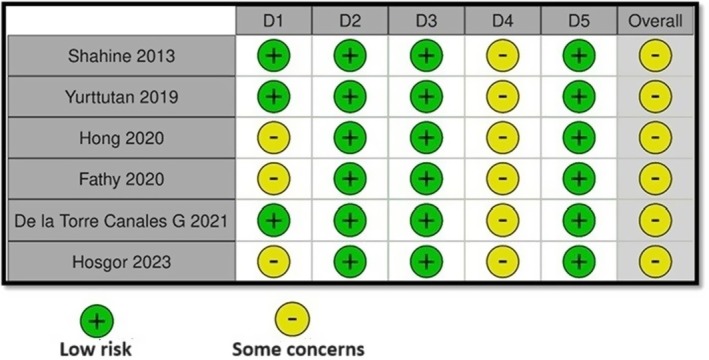
Quality analysis of studies with the RoB2 tool. D1: Bias due to the randomization process. D2: Bias due to deviations from the intended interventions. D3: Bias due to missing outcome data. D4: Bias arising from the measurement of the outcome. D5: Bias due to the selection of the reported outcome. Overall: Overall risk of bias.

**Fig. 3:**
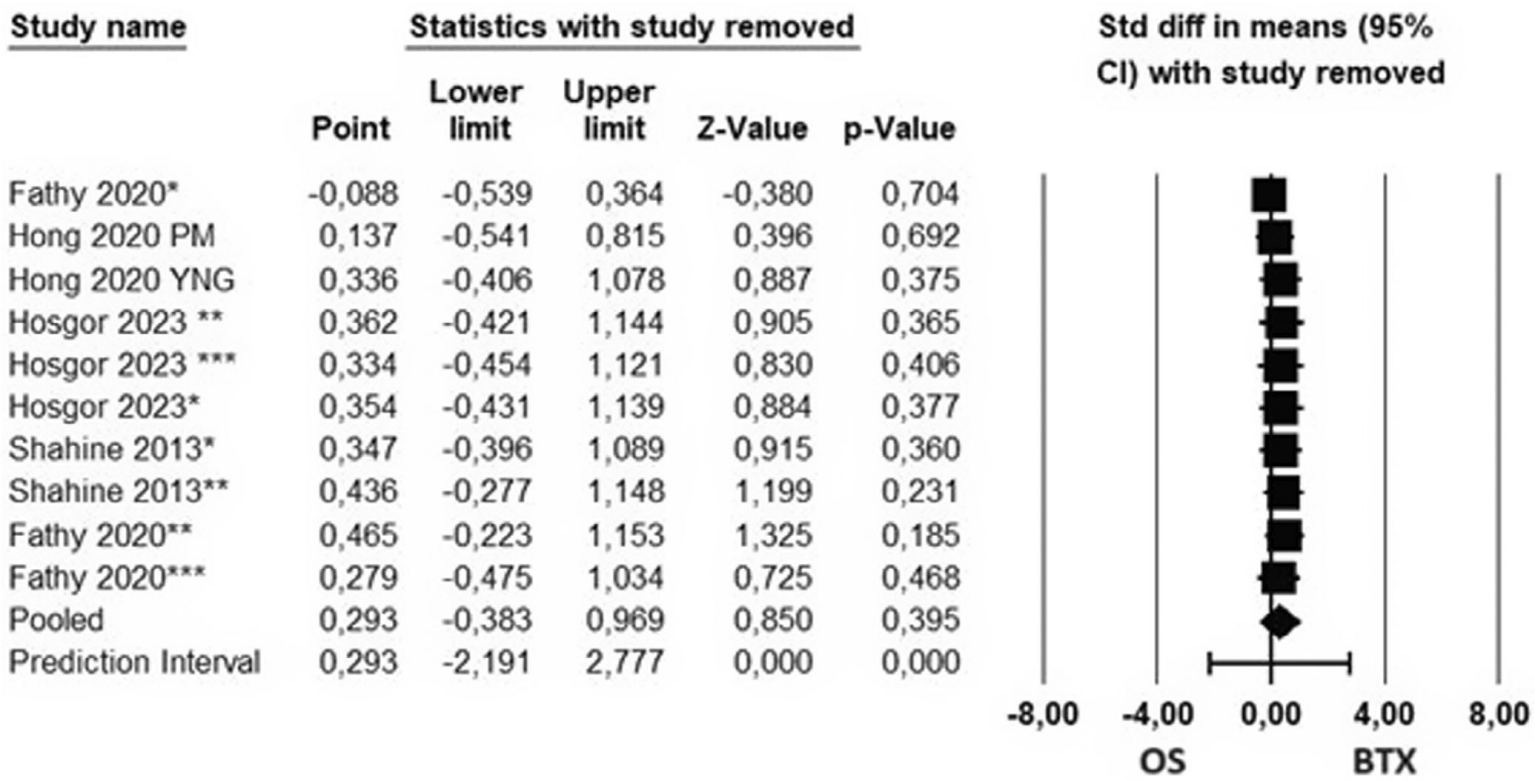
Sensitivity analysis excluding one of the studies in which maximum mouth opening was evaluated. The values indicated opposite the studies in the dot column indicate the total effect size calculated by excluding the relevant study.

**Fig. 4:**
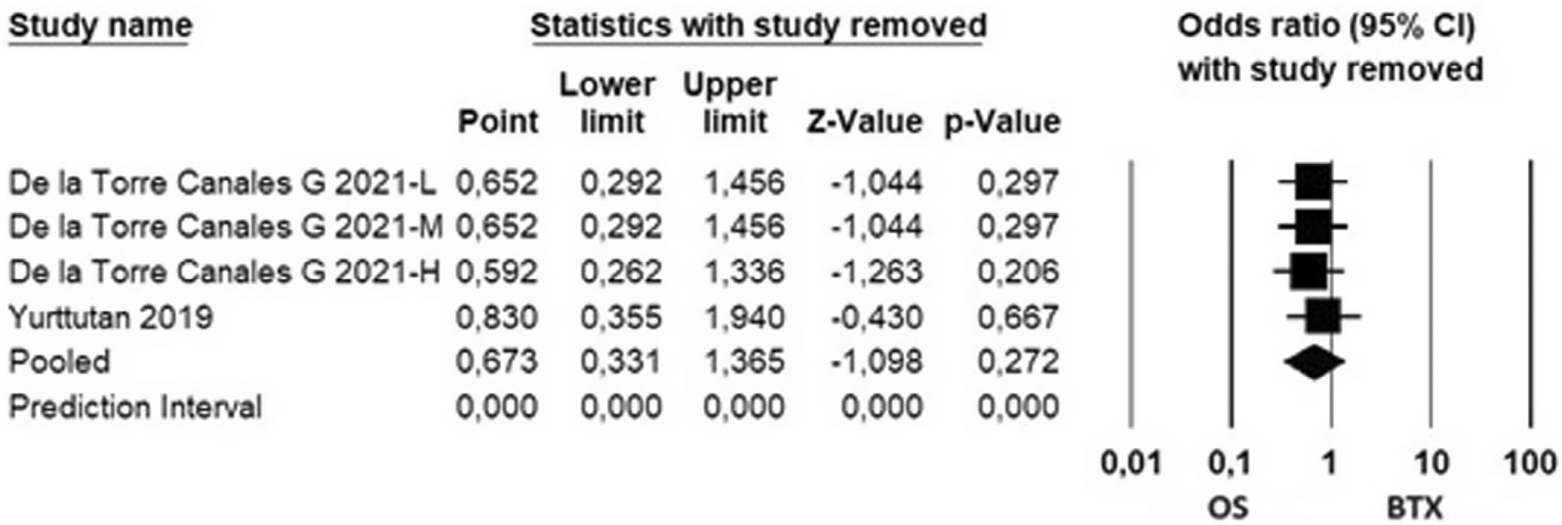
Sensitivity analysis excluding one of the studies evaluating the graded chronic pain scale. The values indicated opposite the studies in the dot column indicate the total effect size calculated by excluding the relevant study.

All studies included in the study were carried out between 2013 and 2023 and included clinical follow‐ups of OS and BTX applied to individuals with temporomandibular disorders. In the clinical follow‐ups in the studies, the parameters of the participants' GCPS and MMO at different times were examined, and these parameters were included in the meta‐analysis. The data obtained from the studies are presented in Tables 1 and 2, and the quality analyses of the studies are given in Fig. 2. In the sensitivity analyses carried out by excluding one study every time, it was observed that the effect sizes did not change significantly (Figs 3 and 4).

**Table 2:** Data extracted from studies evaluating the graded chronic pain scale (GCPS)

Author, year	Mean age ± SD (gender)	Intervention (sample size)		Follow‐up period	GCPS
		BTX	OS		
Yurttutan 2019^28^	OS:31 ± 7.33 (16 women, 9 men) BTX: 30.5 ± 9.95 (15 women, 9 men)	BTX (n = 24) 90 U Botox, Allergan injected into temporal and masseter muscles(15 U into each temporalis muscle and 30 U into each masseter muscle) (Five points injection sites for the masseter muscle and three points injection sites for temporalis muscle)	OS (n = 25) 2 mm autopolymerized hard acrylic resin splint used in the maxillary arch 12 hours a day for 6 months	6 months	BTX T0 GCPS (II–III–IV):11 sixth month GCPS (II–III–IV):2 OS T0 GCPS (II–III–IV):12 sixth month GCPS (II–III–IV):7
De la Torre Canales G 2021^29^	Age not accessible (Women)	BTX‐L (n = 20) Temporal muscle 10 U, masseter muscle 30 U Botox, Allergan were injected (Five injections per muscle during a single appointment)	OS (n = 20) The splint prepared from heat‐polymerized acrylic resin in the maxillary arch was used during sleep during the follow‐up period	6 months	BTX‐L T0 GCPS (II–III–IV):17 sixth month GCPS (II–III–IV):1 OS T0 GCPS (II–III–IV):19 sixth month GCPS (II–III–IV):4
		BTX‐M (n = 20) Temporal muscle 20 U, masseter muscle 50 U Botox, Allergan were injected (Five injections per muscle during a single appointment)			BTX‐M T0 GCPS (II–III–IV):18 sixth month GCPS (II–III–IV):2 OS T0 GCPS (II–III–IV):19 sixth month GCPS (II–III–IV):4
		BTX‐H (n = 20) Temporal muscle 25 U, masseter muscle 75 U Botox, Allergan were injected (Five injections per muscle during a single appointment)			BTX‐H T0 GCPS (II–III–IV):19 sixth month GCPS (II–III–IV):4 OS: T0 GCPS (II–III–IV):19 sixth month GCPS (II–III–IV):4

BTX‐H = high dosage botulinum toxin type A injected group; BTX‐L = low dosage botulinum toxin type A injected group; BTX‐M = medium dosage botulinum toxin type A injected group.

### MMO amounts

Publications examining MMO amount include first‐week follow‐ups as well as first‐, second‐, third‐, sixth‐ and twelfth‐month follow‐ups. When the studies were evaluated independently from the follow‐up period, a heterogeneous structure was observed (*τ* ^2^ = 1.042, *Q* = 88.324, df = 9, *I* ^2^ = 89.81, *P* < 0.001). According to the results of the random effect model, an effect size of 0.293 was detected in favour of BTX, but the amount of MMO did not show a statistically significant difference in the groups treated with OS and BTX (95% CI – 0.383 to 0.969, *P* = 0.395, *z* = −0.850).

**Fig. 5:**
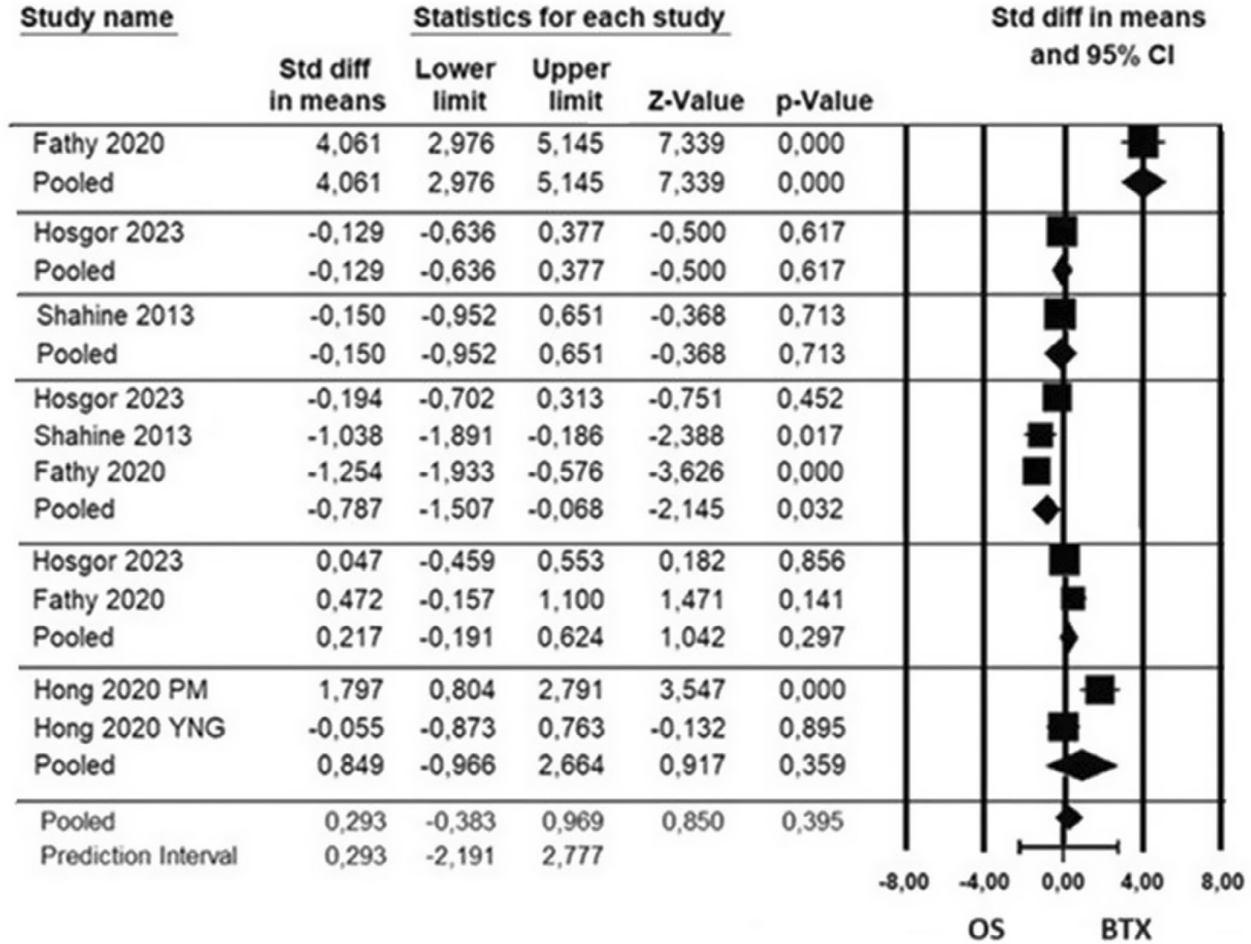
MMO amounts of participants in the studies at first week, first, second, third, sixth and twelfth month. Rows are ordered from the first week to the twelfth month. PM: study with postmenopausal participants, YNG: study with young participants.

Subgroup analyses were carried out based on follow‐up periods to determine the source of heterogeneity, and a statistically significant difference was observed between the effect sizes of the studies (*P* < 0.05). The amounts of MMO were significantly higher in the BTX group in the studies including the follow‐ups in the first week and in the OS group in the studies including the follow‐ups in the third month. In other follow‐up periods, the MMO amount did not differ significantly for OS and BTX groups (*P* > 0.05) (Fig. 5).

### GCPS scores

**Fig. 6:**
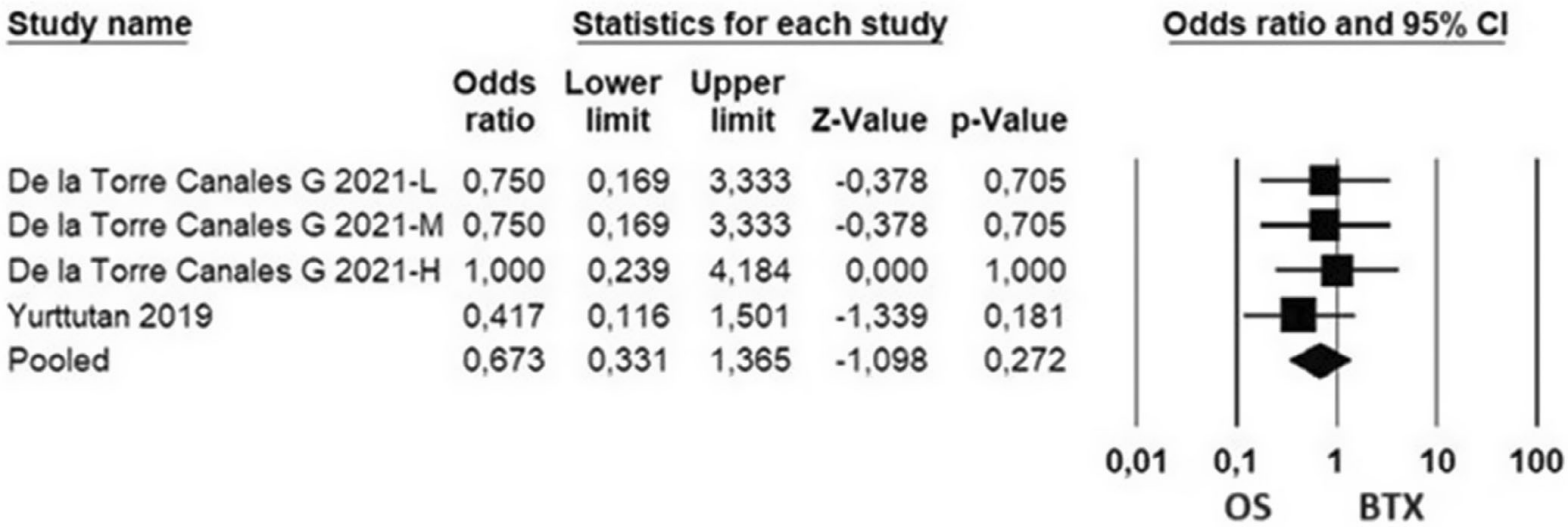
Analyses of GCPS changes of the participants in the studies over a 6‐month period. L: The group was treated with low‐dose BTX. M: The group administered moderate BTX. H: group with a high dose of BTX.

The publications examining the GCPS cover the follow‐ups in the sixth month. While evaluating the studies, the improvement criterion of the participants was based on the decrease of GCPS scores from Levels II, III and IV to Levels 0 or I. There is no pain or interference related to TMD at Level 0, and Level I refers to low pain and no interference. While there is a high degree of pain at Level II, there is no interference. Level III refers to moderate interference and Level IV refers to severe interference. When the relevant publications were evaluated, no heterogeneity was determined (*P* > 0.1). The results of the random effect model showed an effect size of 0.673 in favour of OS (95% CI 0.331 to 1.365, *P* = 0.272, *z* = −1.098); however, GCPSs of OS and BTX did not show a statistically significant difference (*P* > 0.05) (Fig. 6).

## DISCUSSION

This systematic review and meta‐analysis study evaluated the effectiveness of OS and BTX applications in individuals with TMD. The analysis of six studies from PubMed, Web of Science, Scopus and Google Scholar databases revealed the effects of these treatments on MMO amounts and GCPS. These parameters are recognized and reliable measures commonly used to measure pain and functional disorders in TMD.^18^, ^19^

The results of the study can guide the development of more effective strategies in the treatment of TMD by helping to understand which OS and BTX application used in the treatment of TMD is more effective.

Considering the MMO amounts, subgroup analyses by follow‐up periods showed a time‐varying effect on treatment effectiveness. While the amount of MMO was significantly higher in the BTX group in the first‐week follow‐up, higher MMO amounts were detected in the OS group in the third‐month follow‐up. This result suggests that BTX provides better functional improvement in the short term and OS in the long term. This situation is consistent with the pharmacological properties of BTX, which inhibits acetylcholine release at the neuromuscular junction and causes transient muscle paralysis lasting about 3–4 months.^20^ On the other hand, OS treatment is a mechanical device aimed at stabilizing occlusion, reducing muscle hyperactivity and joint loading. In addition, it might have a more gradual but longer lasting effect.^1^ Although there was a significant difference between the treatment efficiencies in the first‐week and third‐month follow‐up periods, the absence of any difference in the first‐ and second‐month follow‐ups indicates that the effectiveness might change over time and different treatment methods might be superior at certain time points, and in this context, the results of the study might help to make the treatment processes of individuals with TMD more effectively. The results also reveal that there was no significant difference between OS and BTX treatments at the sixth‐ and twelfth‐month follow‐ups, indicating that both treatments had similar long‐term effects on the amount of MMO. When the GCPS scores in the sixth month were examined, it was seen that both treatment processes similarly relieved chronic pain. This suggests that both OS and BTX treatments have similar effects on pain and function in the long term. The results of the study are consistent with previous studies evaluating the effects of OS and BTX treatments on TMD.^21^, ^22^, ^23^ At this point, it is important to note that each treatment method has its own advantages and disadvantages. Although OS applications and BTX injections provide similar results, they work with different mechanisms and might therefore be more appropriate in different situations. Generally, OS applications are less invasive and have fewer side effects. However, the application of this treatment usually during the night can be uncomfortable for some patients. On the other hand, BTX injections can provide faster and more pronounced results, but there is a possibility of side effects in some patients since they are a more invasive treatment method. When deciding which treatment method to apply, the patient's condition and needs should be taken into consideration. Factors such as the patient's pain level, lifestyle, general health status and tolerance to treatment play an important role in terms of determining which treatment method will be more appropriate. Therefore, when making a treatment plan, it is important to adopt a specific approach to the patient's condition and needs. This will help the patient to obtain the best results and maximize the quality of life.

This meta‐analysis study has certain limitations. First of all, the inadequacy of the terms used in the databases to search for trials might result in some publications being overlooked. In addition number of studies included in the meta‐analysis and the sample sizes of these studies are limited, and there are some concerns about the quality of the publications according to the quality analysis performed with the RoB 2 tool. This might affect the general validity and reliability of the analysis results. Another limitation is the use of different BTX types and doses in the studies included in the meta‐analysis. This might make it difficult to compare the effectiveness of different treatment protocols and reduce the reliability of the analysis results. Moreover, the effects of OS and BTX on other clinical parameters such as joint sounds, bruxism and quality of life of individuals were not compared in this study. This limits the scope of the analysis and might limit the clinical applicability of the analysis results by ignoring some important clinical results.

## CONCLUSION

It is observed that the effects of OS and BTX treatments on pain and function in individuals with TMD are complex and variable over time. While different treatment methods might have advantages in the first‐week and third‐month follow‐ups, both treatment methods provide similar results in the long term. Therefore, individual patient needs and preferences can be taken into consideration when choosing the treatment method. Due to certain limitations of the meta‐analysis study, these results cannot be considered to be precise. Therefore, more randomized controlled trials are needed to better understand the role of OS and BTX in the treatment of TMD. Furthermore, the effects of OS and BTX on other clinical parameters of TMD should also be investigated.

## AUTHOR CONTRIBUTIONS

**Ö İşisağ:** Conceptualization; methodology; validation; investigation; writing – original draft. **H Atasoy:** Writing – original draft; methodology; validation. **S Yıldız:** Conceptualization; methodology; validation.

## FUNDING INFORMATION

There was no funding source for this study.

## CONFLICT OF INTEREST STATEMENT

The authors declare no competing interests.

## ETHICS STATEMENT

For this type of study, ethical approval was not required. Informed consent is not applicable for this type of study.

## Data Availability

The data for this research can be found in Tables 1 and 2, and the same data can be found in the available articles from references.^24–29^
